# Study of Different Local Treatments of Post COVID-19 Smell Dysfunction 

**DOI:** 10.22038/IJORL.2022.58339.3012

**Published:** 2022-11

**Authors:** Soad A. Mohamad, Ahmed M. Badawi, Ramy M. El-Sabaa, Hosam M. Ahmad, Asmaa S. Mohamed

**Affiliations:** 1 *Department of Pharmaceutics and Clinical Pharmacy, Faculty of Pharmacy, Deraya University, Minia, Egypt.*; 2 *Department of Otorhinolaryngology, Faculty of Medicine, Minia University, Minia, Egypt.*; 3 *Department of Clinical Pharmacy, Faculty of Pharmacy, Menoufia and Deraya Universities, Egypt.*; 4 *Departments of Internal Medicine and Biomedical Chemistry, Egypt Ministry of Health and population, Minia, Egypt.*; 5 *Department of Clinical Pharmacy, Faculty of Pharmacy, Port Said University, Port said, Egypt. *

**Keywords:** Antihistamines, Butanol threshold test, Corticosteroids, Discrimination test, Smell dysfunction, Post COVID-19

## Abstract

**Introduction:**

This study was designed to differentiate between the impact of the topical nasal spray of corticosteroids, antihistamines, a combination of them, and normal 0.2% saline in treating patients with post-coronavirus disease 2019 (COVID-19) smell dysfunction.

**Materials and Methods:**

Patients with hyposmia or anosmia (n = 240), who recently recovered from COVID-19, were enrolled in this trial and were randomly assigned to four parallel groups. Group I (G1) received a combination of topical corticosteroid and antihistamine nasal spray (n = 60). Group II (G2) received topical corticosteroid nasal spray (n = 60). Group III (G3) received antihistamine nasal spray (n = 60). Group IV (G4) received 0.2% normal nasal saline nasal spray (n = 60). The treatments were used in all groups for 3 weeks. The sense of smell was assessed using the butanol threshold and discrimination tests. The smell tests were evaluated weekly for 3 weeks.

**Results:**

The mean age of the patients was 51.9 ± 7.1 years; moreover, 83.8% and 16.2% were male and female, respectively. The results of the smell tests in the first week significantly improved with those in the third week (P< 0.001). The greatest degree of improvement was found in the first group, followed by the second, third, and fourth groups.

**Conclusions:**

The results suggest the ability of combination therapy of corticosteroid and antihistamine nasal spray to manage post-COVID-19 hyposmia or anosmia; however, this combination therapy was not superior to corticosteroid nasal spray. Trial registration ID: UMIN000043537.

## Introduction

Since the beginning of the coronavirus disease 2019 (COVID-19) pandemic in China (2019), the symptoms of the disease became clear like fever, cough, fatigue, and myalgia. Some cases had mild or severe pneumonia (1). Other symptoms, such as allergies and loss of the sense of smell (anosmia) and taste (ageusia), may also occur (2). It was found that anosmia persists in these patients even after recovery for weeks to months, which decreases the quality of life of these patients (3,4). The reason for this can be traced back to the existence of a high viral load within the nasal cavity (5). 

Olfactory dysfunction may indicate an immunoinflammatory response or peripheral injury of the first cranial nerve (6). Anosmia in patients with COVID-19 is caused by three pathways, according to many studies: 1) local infection of support cells and vascular pericytes in the nose (conductive type); 2) damage to sensory epithelial support cells; and 3) destruction of sustentacular cells and Bowman’s gland cells (sensorineural types) (7). According to guidelines, systemic steroids may be effective in treating olfactory dysfunction after COVID-19 (2). Local corticosteroids can improve olfactory function by altering olfactory receptor neurons at a dose of 200 µg of mometasone furoate (MF) or 110 µg of fluticasone furoate (FF), both administered in the morning for 4 weeks (8). 

Second-generation oral antihistamines (e.g., desloratadine, fexofenadine, loratadine, and cetirizine) are the first option recommended for treating all patients with allergic rhinitis. Antihistamines are widely used for treating different olfactory dysfunctions, particularly second-generation antihistamines, because of their high safety and efficacy (9,10). This study was designed to identify the effectiveness of topical steroids and antihistamines in treating post-COVID-19 hyposmia or anosmia.

## Materials and Methods

This was a single-center, active placebo controlled study. The study duration was from January 1, 2021 to February 28, 2021. This study was conducted according to the Declaration of Helsinki. The study protocol and consent procedure received ethical approval from the Ethics Committee of Minia University (approval no.69). All participants provided documented informed consent before participating in this study.

Patients were randomly assigned to one of four parallel groups, initially in a 1:1:1:1 ratio. Patient selection and allocation were performed by simple randomization using a computer-generated list of random numbers. This was a triple-blind study as all study personnel, patients, researchers, and other staff involved in the study (e.g., data collectors and statisticians) were blinded to group assignment and treatment allocation. Allocation concealment was performed using sealed opaque envelopes for the different treatments. All selected patients were previously diagnosed with mild or moderate (non-hospitalized) COVID-19, according to the management protocol for patients with COVID-19 in Egypt (11).

The study population included adult outpatients (non-hospitalized) who were attending the ear, nose, and throat clinic at Minia University Hospital monthly and who recently recovered from proven COVID-19 infection (the duration from COVID-19 recovery to the second negative real-time reverse transcription-polymerase chain reaction (rRT-PCR) sample does not exceed 1 week) and who had been complaining of hyposmia or anosmia. The recovery was defined as two consecutive negative rRT-PCR samples, and the time interval between the two samples was 48 h. The minimum sample size required was calculated using a sample size calculator program with a confidence level of 95% and a standard error of 0.05. Proper randomization, blinding, and the use of a placebo were performed to control confounding factors and bias. Initially, 240 patients were enrolled in this study; however, only 207 patients participated and completed the study. The inclusion criteria were as follows: patients aged ≥ 40 years, those who recently recovered from proven COVID-19 infection (the duration from COVID-19 recovery to the second negative rRT-PCR sample does not exceed 1 week), and those who had been complaining from hyposmia or anosmia. The diagnosis of COVID-19 was confirmed using rRT-PCR and chest radiology. All selected patients were previously diagnosed with mild or moderate (non-hospitalized) COVID-19. The exclusion criteria were as follows: patients with diabetes mellitus (DM), those with hypertension (HTN), smokers, those with a history of neurological disease that can affect smell sensation, those who received topical nasal steroids or antihistamines or saline, those who have used systemic steroids or antihistamines, those with previous chronic rhinologic pathologies, those whose anosmia improved before COVID-19 recovery, pregnant women, and those who did not agree to participate in the study. Demographic data were obtained from the patients, including age, sex, the duration of smell dysfunction, the severity of COVID-19 illness, and the period from COVID-19 recovery to the last negative rRT-PCR sample. Physical examination (including nasal endoscopy and rhinoscopy) was performed aseptically. Then, the patients included in this study were randomly allocated into four groups: Group I (G1) included 60 patients who received a combination of topical corticosteroids and antihistamine nasal spray (azelastine base/ fluticasone propionate) 125 µg/50 µg/25 mL actuation nasal spray, 120 metered sprays, one puff in each nostril twice daily. Group II (G2) included 60 patients who received topical corticosteroids (aqueous suspension of microfine fluticasone propionate for topical administration to the nasal mucosa using a metering, atomizing spray pump). 

Fluticasone propionate was prepared in 50 µ/100 mg of spray supplied by the nasal adaptor. The dose was one puff in each nostril twice daily. Group III (G3) included 60 patients who received antihistamines (azelastine HCl nasal spray containing 125 µg of azelastine base) 1 puff in each nostril twice daily. Group IV (G4) included 60 patients who received 0.2% normal nasal saline, one puff in each nostril every 4 h as the control group. The primary outcome evaluated in this study was the patients’ sense of smell, which was assessed using the butanol threshold (12) and discrimination (13) tests. All patients were initially evaluated after their recovery from COVID-19 and were followed up for 3 weeks. The scores of the aforementioned smell tests were recorded weekly.

***Butanol threshold test:***

For each trial, two glass bottles were presented to the subject. There was water in one and a diluted concentration of 4% butanol in deionized water in the other. Possible scores ranged from 0 to 9. The highest concentration of butanol was in bottle 0; every subsequent dilution (bottles 1–9) was 1:9 dilutions with deionized water. The bottles of butanol were ordered ascending from the lower concentration of butanol to the highest one in alternative to water. The two glass bottles were similar in size and appearance. The patients were instructed to occlude one nostril and smell the tip of the first bottle with the other nostril. Then, the same procedure was performed for the second bottle, and the patients must choose which of the bottles did not contain water (12). The test was repeated until the first concentration that the patient could distinguish. Then, the mean of the scores for the two nostrils was calculated.

***Smell discrimination test:***

In this test, triplets of two odorous substances (e.g., coffee) and one odorless substance (i.e., water) were used for each nose. The participants were instructed to distinguish the bottles that contained odorous substances from the one that contained water. The odorous substances used must meet the following criteria: they must be familiar and well known to the participants and should be similar in both intensity and hedonic tone (13). The patients were instructed to occlude the untested nostril. To avoid visual detection of the substances, the participants were blindfolded with a hygienic facemask. To save time, the participants were permitted to sniff each bottle only once. In a fixed randomized order, the three bottles were submitted to each nostril with 16 trials. If the participants answered correctly, the bottles were used in triplicate for each trial, and a score was given. For each nostril, the score ranged from 0 to 16. Then, the mean of the scores for the two nostrils was calculated. Statistical analysis: The data obtained were analyzed using Statistical Package for the Social Sciences, version 26. A normality test was applied to all data. Descriptive analysis was used to summarize the data. The comparison between two paired groups with quantitative data and parametric distribution was performed using the paired t-test. 

The chi-square test was used to compare two groups with qualitative data. The confidence interval was set at 95%, and the margin of error accepted was set at 5%. One-way analysis of variance was used to compare the quantitative data between the four groups, followed by Tukey’s post hoc test between every two groups. A p-value of less than 0.05 was used to denote statistical significance.

**Fig 1:**
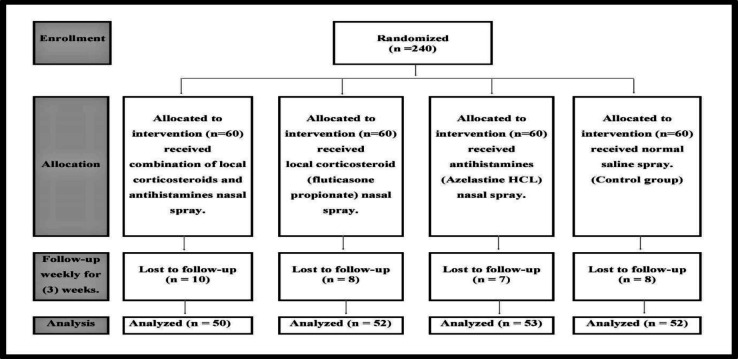
Flow Diagram of the study

Figure 1 shows that 240 participants were enrolled in this study. However, after 3 weeks, only 207 participants were followed up because of several reasons, including partial or non-compliance to treatment, non-response, loss of contact, and consent withdrawal.

## Results

The sample under study consisted of 240 participants, divided into four groups with 60 participants in each group. 

The number of male participants was 201 (83.8%), and the number of female participants was 39 (16.2%). According to residence, 223 participants (92.9%) were living in urban areas, whereas 17 participants (7.1%) were from rural areas. No significant differences in the duration from COVID-19 recovery to the last negative rRT-PCR sample and baseline smell tests’ results (butanol and discrimination tests) were observed between the four groups (Table 1).

**Table 1:** Baseline demographic characteristics of the four groups

			**Group**				**P**
			**Combination** **(G1)**	**Corticosteroids** **(G2)**	**Antihistamines** **(G3)**	**Saline** **(G4)**	
Age (Mean ±SD)			52.65±7.67	51.90±7.25	50.85±6.74	51.98±6.95	0.59
Sex	Male	Count	50	51	54	46	0.26
		%	83.3%	85%	90%	76.7%	
	Female	Count	10	9	6	14	
		%	16.7%	15%	10%	23.3%	
Residence	Urban	Count	55	59	55	54	0.29
		%	91.7%	98.3%	91.7%	90%	
	Rural	Count	5	1	5	6	
		%	8.3%	1.7%	8.3%	10%	
Duration of smell dysfunction (days) (M±SD)			10.7±3.4	10.9±2.9	10.8±3	10.3±3.1	0.71
The duration from COVID-19 recovery (days) (M±SD)			3.55±1.77	3.47±1.7	3.32±1.81	3.27±1.76	0.8
Butanol test (1) (M±SD)			3.59±1.28	3.84±1.23	3.8±1.36	3.88±1.19	0.6
Discrimination test (1) (M±SD)			5.41±2.3	5.82±2.08	5.78±2.41	5.85±2.14	0.68

Table 1 shows no significant difference in the baseline demographic characteristics between 

the four groups.

**Fig 2:**
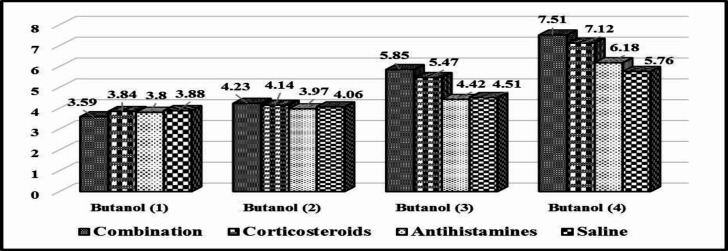
The progress in butanol threshold test’s scores between different groups over the 3 weeks of follow-up

Figure 2 shows the gradual increase (improvement) in the butanol threshold test’s scores over the 3-weeks follow-up period. An increase in the scores in the first, second, third, and fourth groups was observed respectively.

**Fig 3:**
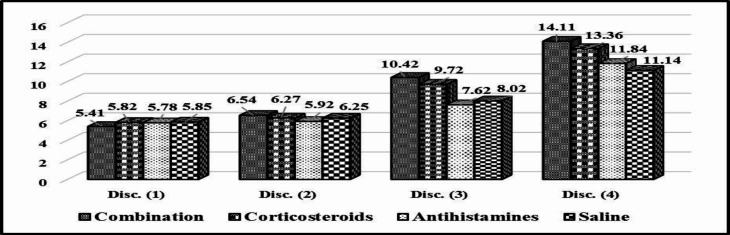
The progress in discrimination test’s scores between different groups over the 3 weeks of follow-up

Figure 3 shows the gradual increase (improvement) in the discrimination test’s scores over the 3-weeks follow-up period. An increase in the scores in the first, second, third, and fourth groups was observed respectively.

**Fig 4:**
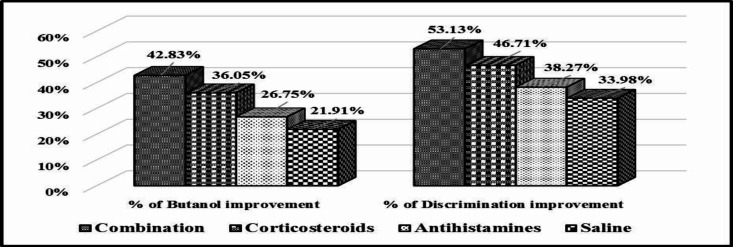
Percent of butanol threshold and discrimination tests’ scores improvement after treatment with the different topical drugs in comparison to the control

Figure 4 shows that the best results were observed in the first group, followed by the second, third, and fourth groups, in this particular order.

**Table 2:** Statistical differences in scores between the four groups before and after treatment (3 weeks)

**Group**		**Butanol test (1)**	**Butanol test (4)**	**P** **value**	**Disc. ** **test (1)**	**Disc. ** **test (4)**	**P** **value**
Combination (G1)	Mean	3.59	7.51	<0.001*	5.41	14.11	<0.001*
	SD	1.28	1.35		2.3	2.02	
Cortisone (G2)	Mean	3.84	7.12	<0.001*	5.82	13.36	<0.001*
	SD	1.23	1.23		2.08	2.15	
Antihistamines (G3)	Mean	3.8	6.18	<0.001*	5.78	11.84	<0.001*
	SD	1.36	1.34		2.41	2.52	
Saline (G4)	Mean	3.88	5.76	<0.001*	5.85	11.14	<0.001*
	SD	1.19	1.44		2.14	2.48	

Butanol (1) = baseline butanol test’s score. Butanol (4) = follow-up butanol test’s score after the third week.

Disc. (1) = baseline discrimination test’s score. Disc. (4) = follow-up discrimination test’s score after the third week.

The paired samples t-test was performed to compare the mean scores before and after treatment.

It was obvious from the results shown in Table 2 that there was a significant improvement in the test’s scores in G1 and G2 after 3 weeks of treatment. In the four groups, a statistically significant increase (improvement) in the tests’ results was observed in the first and third weeks.

**Table 3:** Comparison between the results of the butanol threshold and discrimination tests

**Butanol test (4)**					
	**Combination (G1)**	**Corticosteroids (G2)**	**Antihistamines (G3) **	**Saline (G4)**	**P value**
(M±SD)	7.51±1.35	7.12±1.23	6.18±1.34	5.76±1.44	< 0.001*
P value (multiple comparisons)					
G1 vs. G2	0.11	G1 vs. G4	<0.001*	G2 vs. G4	<0.001*
G1 vs. G3	<0.001*	G2 vs. G3	<0.001*	G3 vs. G4	0.118
**Discrimination test (4)**					
	Combination (G1)	Corticosteroids (G2)	Antihistamines (G3)	Saline (G4)	P value
(M±SD)	14.11±2.02	13.36±2.15	11.84±2.52	11.14±2.48	< 0.001*
**P value (multiple comparisons)**					
G1 vs. G2	0.098	G1 vs. G4	<0.001*	G2 vs. G4	<0.001*
G1 vs. G3	<0.001*	G2 vs. G3	0.001*	G3 vs. G4	0.117

One-way analysis of variance was used to compare the mean scores of the tests between the four groups, followed by Tukey’s post hoc test between every two groups.

It can be concluded from Table 3 that the two tests used in the trial were accurately designed and enforced on the patients enrolled. Therefore, the tests statistically proved the ability of corticosteroids alone (G2) or in combination (G1) to improve post-COVID-19 smell disorders, without significant differences between the two groups. Additionally, it was statistically proven that antihistamines alone (G3) have a minor role in improving post-COVID-19 smell disorders, without significant differences between antihistamines and saline.

## Discussion

Olfactory disturbances in patients with COVID-19 are frequent and common in the late stages, despite recovery. Understanding the pattern of COVID-19 viral load in tissues is critical for developing future treatment strategies. Until now, no definitive treatment recommendations exist for post-viral patients with olfactory or gustatory impairment, including COVID-19.

Physicians frequently use oral steroids to treat anosmia, with the objective of reducing inflammation and edema. Simultaneously, others oppose the use of systemic corticosteroids for their dangerous drawbacks, particularly when used over a long period (14). 

Local intranasal steroids have several advantages over systemic steroids, including the ease of use, local effect, low systemically absorbed dosage, and good compliance for the patient. Intranasal steroids are unlikely to be harmful; however, we wanted to determine their efficiency and safety in these patients.

This prospective study was designed to examine the efficacy of different patterns of local treatments in improving post-COVID-19 smell dysfunction, assuming high safety levels. In this study, we evaluated the efficacy of intranasal corticosteroids and antihistamines for treating anosmia in patients who recently recovered from COVID-19.

The results revealed that the combination of local steroids and antihistamines improved the scores of the participants in the butanol (7.55 ± 1.35) and discrimination (14.11 ± 2.02) tests after 3 weeks, followed by nasal steroids alone where the scores in both tests improved by 7.12 ± 1.23 and 13.34 ± 2.15, respectively, after 3 weeks. Additionally, Sivam et al. examined the effects of MF administered for 2 weeks on inflammation in the olfactory region and olfactory loss in patients with seasonal allergic rhinitis and reported that this treatment modality improved nasal symptoms, reduced nasal inflammation due to reduced eosinophilic inflammation in the olfactory region, and improved symptoms of allergic rhinitis. The presence of eosinophils in the olfactory area in seasonal allergic rhinitis may indicate that inflammation has a direct, harmful effect on the olfactory epithelium in this disease (15).

Baradaranfar, M.H. et al. examined the efficacy of fluticasone propionate nasal spray in restoring the smell function in patients with nasal polyposis. Thirty patients with hyposmia or anosmia were assessed after medical treatment with fluticasone propionate nasal spray for 8 weeks (400 µg bd). Olfactory assessment was performed eight and 12 weeks after the treatment course, the rate of complete remission was 20%, and six patients experienced complete remission. The sense of smell was impaired in one patient (3.33%) only after treatment (16). Another study found that using MF nasal spray as a topical corticosteroid treatment to treat post-COVID-19 anosmia provides no advantages over olfactory training in terms of smell ratings, the duration of anosmia, and the recovery rates (17). A prospective study found improvements in the odor threshold and identification smell tests after treatment with steroid nasal drops in patients with chronic rhinosinusitis with nasal polyps (18). In contrast, a study examined the effects of topical steroids and reported no statistically significant difference between the treated and untreated nostrils (19). Recently, a study showed that mast cell activation syndrome that remained after COVID-19 illness was responsible for inducing inflammation and allergic-type issues, including biogenic amines, proteases, cytokines, and eicosanoids (20). 

A recent study showed that the use of antihistamines may help minimize the histamine-mediated cytokine storm and recommended the use of antihistamines in the treatment protocol for COVID-19 (21). 

A randomized double-blind trial demonstrated that antihistamines significantly improved the smell scores of olfactory dysfunction (22). In contrast, anosmia did not improve, and the results confirmed that antihistamines alone are statistically a weak competitor for improving the sense of smell after COVID-19 (23). 

In this study, nasal saline was used as the control because nasal saline irrigation is a common treatment for sino-nasal conditions. Another study reported that nasal saline irrigation (16.7%) was used to treat olfactory dysfunction (24). 

The limitations of this study included the exclusion of patients positive COVID‐19, the higher proportion of male respondents, loss of follow‐up and PCR tests for COVID‐19, which are highly sensitive and highly specific, although a false-negative test result can happen in a very low viral load.

## Conclusion

Olfactory dysfunction is considered a significant symptom in the clinical presentation of COVID-19 infection. The recovery of olfactory function in patients with COVID‐19 is valuable to improve quality of life. According to this study, a combination of local corticosteroids and antihistamines has a superior effect over antihistamines alone and nasal saline, followed by local corticosteroids alone, in treating post-COVID-19 hyposmia or anosmia. The results cannot be generalized because of the presence of some limitations; therefore, future clinical studies are needed to explain the mechanisms underlying the development of local treatment.
